# Heparin acts as a structural component of β-endorphin amyloid fibrils rather than a simple aggregation promoter†
†Electronic supplementary information (ESI) available. See DOI: 10.1039/c6cc09770g



**DOI:** 10.1039/c6cc09770g

**Published:** 2016-12-21

**Authors:** N. Nespovitaya, P. Mahou, R. F. Laine, G. S. Kaminski Schierle, C. F. Kaminski

**Affiliations:** a Laser Analytics Group , Department of Chemical Engineering and Biotechnology , Cambridge University , Pembroke Street , Cambridge , CB2 3RA , UK . Email: cfk23@cam.ac.uk

## Abstract

The aggregation promoter heparin is commonly used to study the aggregation kinetics and biophysical properties of protein amyloids. However, the underlying mechanism for amyloid promotion by heparin remains poorly understood. In the case of the neuropeptide β-endorphin that can reversibly adopt a functional amyloid form in nature, aggregation in the presence of heparin leads to a loss of function. Applying correlative optical super-resolution microscopy methods, we show that heparin incorporates into emerging β-endorphin fibrils forming an integral component and is essential for amyloid templating. This will have direct implications on β-endorphin's normal physiological function and raises concerns on the biological relevance of heparin-promoted amyloid models.

## Abstract 2

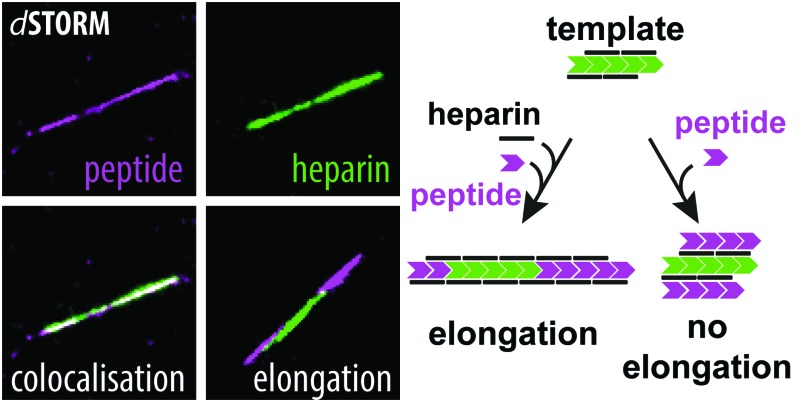
Super-resolution microscopy gives molecular level insights into the heparin-promoted aggregation of β-endorphin amyloid fibrils.

The endogenous opiate β-endorphin is an essential neuropeptide involved in the stress response of higher organisms.^1^ In the secretory pathway, it is temporarily stored in dense-core vesicles in the amyloid form, which disaggregates upon vesicle secretion into the blood.^2,3^ The reversibility of aggregation of β-endorphin and some other neuropeptides renders them fundamentally different from most functional amyloids, and probably all disease-related amyloids, which aggregate irreversibly.^2–5^

Previous work on β-endorphin aggregation investigated structure–activity relationships of amyloids induced by high- and low-molecular-weight aggregation promoters, including heparin and salts of polyprotic acids.^3^

Heparin is one of the most commonly used promoters of aggregation. However, its mechanism of action is a subject of considerable debate.^6^ Studies with transmission electron microscopy (TEM), fluorescence and NMR spectroscopy revealed morphological and structural differences between β-endorphin aggregates induced with heparin, and those that were not (see Fig. 1). Moreover, heparin-promoted amyloids demonstrated an increased stability in dissociation assays.^3^ These dramatic changes in fibril structure and function raise fundamental questions on the role of heparin in protein aggregation.

**Fig. 1:**
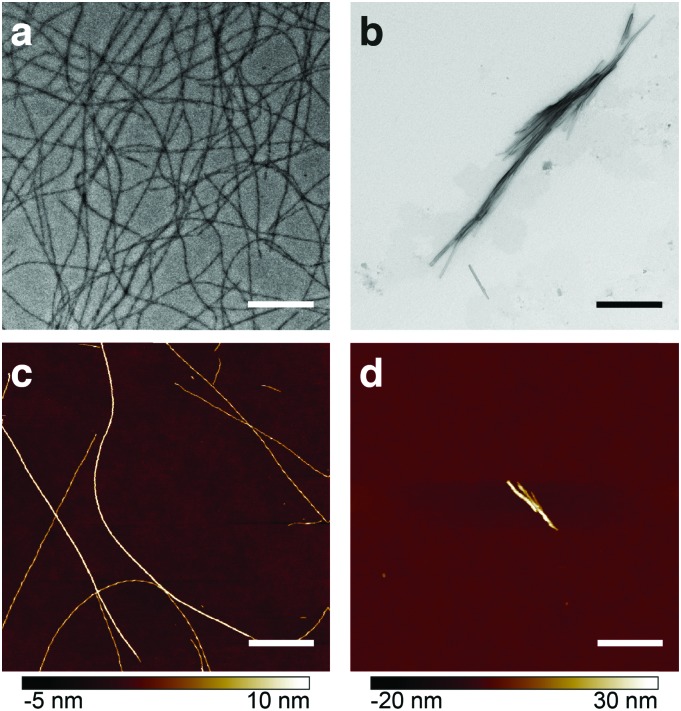
Comparative visualisation of β-endorphin amyloid fibrils obtained in the presence (a and c) and absence of heparin (b and d). TEM (a and b) and AFM (c and d) images show heparin-promoted fibrils as long individual filaments, while β-endorphin amyloids formed in heparin-free aggregation buffer are short and form bundles. In all case, fibrils were obtained at 60 μM peptide concentration (a–d) and 20 μM heparin (a and c). In all cases, aggregation buffer was composed of 50 mM sodium phosphate and 2 mM sodium citrate at pH 6.0. Scale bars are 1 μm.

Our working hypothesis in the present study is that heparin is not simply a molecular crowding agent, as suggested in recent models,^7,8^ but instead that it co-aggregates with the peptide into a composite amyloid, at least in the case of β-endorphin. This, in turn, leads to a change of the biophysical, dynamic structural properties of the β-endorphin amyloid and its morphology. We thus predicted for heparin to be associated with the peptide along the whole fibril axis and tested this hypothesis with two-colour optical super-resolution microscopy (SRM).^9^ In combination with differential labelling, SRM permitted an unambiguous identification of the labelled compounds and their relative arrangement at a nanometric scale.^9–11^ Importantly, alternative high-resolution methods of structural biology, such as NMR spectroscopy and X-ray crystallography, cannot address the question of peptide-heparin colocalisation, due to the structural heterogeneity of natural heparin.^12^ Alternative high-resolution microscopy method such as TEM used in combination with immunogold labelling overcomes that latter disadvantage, however, has comparatively low resolution because of the large sizes of gold particles and linker lengths.^13^ The technique is also hampered by the sparse labelling density usually achieved. Moreover, it does not permit the reliable differentiation of *de novo* fibril growth from the original seed species.

Using two-colour direct stochastic optical reconstruction microscopy (dSTORM)^14^ we made two key findings: (1) heparin co-aggregates with β-endorphin and becomes a structural element of the amyloid, and (2) heparin-promoted fibrils are not capable of templating the amyloid growth in the absence of heparin.

In order to test our hypothesis of colocalisation of β-endorphin and heparin within the fibrils, we obtained β-endorphin amyloids by co-incubation of the peptide labelled with AlexaFluor 568 dye (AF568) and heparin conjugated with AlexaFluor 647 (AF647) and imaged them with two-colour dSTORM. The fibrillar nature of the obtained sample was confirmed by atomic force microscopy (AFM, Fig. 2a). Further, 350 fibrils identified in 35 random fields of view showed colocalisation of fluorescent signals in both the peptide and heparin imaging channels (exemplified in Fig. 2b–d). A pixel-based analysis of β-endorphin and heparin colocalisation within the amyloid fibrils further supports this visual observation (ESI,† Fig. S1). To ensure that fluorescence images were good representations of β-endorphin fibrils, we used stimulated emission depletion (STED)^15^ microscopy correlated with AFM. β-Endorphin fibrils labelled with AF647 attached to either the peptide (Fig. 2e and f) or to heparin (Fig. 2g and h) were imaged with STED and then with AFM. All structures observed by AFM had fibrillar shapes and exhibited the fluorescence signal everywhere along the fibril axis, confirming that all optical images were adequate representations of β-endorphin fibrils and that heparin was evenly distributed along the fibrils. The data obtained here by two independent SRM techniques provide unequivocal evidence of the tight association of heparin with the β-endorphin amyloid fibrils. Next, we tested whether heparin becomes an integral component of β-endorphin fibrils or associates unspecifically with the amyloid. We performed an elongation assay where fibrillar seeds of β-endorphin amyloids were incubated with the monomeric peptide under various conditions (see Fig. 3 cartoons). Two-colour dSTORM demonstrated that heparin-promoted β-endorphin amyloids grow by incorporation of both the peptide and heparin into the fibril termini (Fig. 3b–d). At the same time, heparin-promoted fibril seeds incubated with monomeric β-endorphin in the heparin-free environment did not elongate axially and appeared as short two-colour fibrils (Fig. 3e, f and ESI,† Fig. S2), which suggests lateral adhesion of the added monomer on the seed surface. This notion is supported by a comparative AFM analysis of fibril height before and after the elongation assay, which indeed revealed that the seeds thicken upon the addition of the monomeric peptide (ESI,† Fig. S3). Notably, β-endorphin amyloids elongated in the presence of heparin retained twisted fibrillar morphology similar to parental heparin-promoted seeds (ESI,† Fig. S4).

**Fig. 2:**
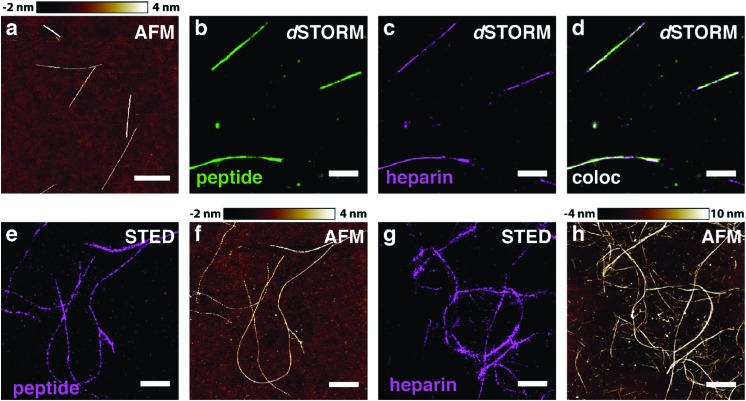
β-Endorphin and heparin are associated within amyloid fibrils. The fibrillar nature of heparin-promoted β-endorphin aggregates labelled with two fluorophores was confirmed by AFM (a). The fibrils were obtained at 60 μM total peptide concentration including 10% (mol) of the AF568-labelled peptide (b, green) and 20 μM heparin labelled with AF647 (c, magenta). Colocalisation is displayed in (d, white), where super-resolved images from (b, green) and (c, magenta) are merged. Further, β-endorphin fibrils containing AF647 fluorophore attached either to the peptide (e and f) or to heparin (g and h) were examined by correlated STED/AFM microscopy. A field of view was first acquired *via* STED (e and g) and subsequently probed by AFM (f and h). Optical images are seemed to fully reflect the fibrillar morphology of β-endorphin amyloids with either labelling scheme. Scale bars are 1 μm in a–d and 2 μm in e–h.

**Fig. 3:**
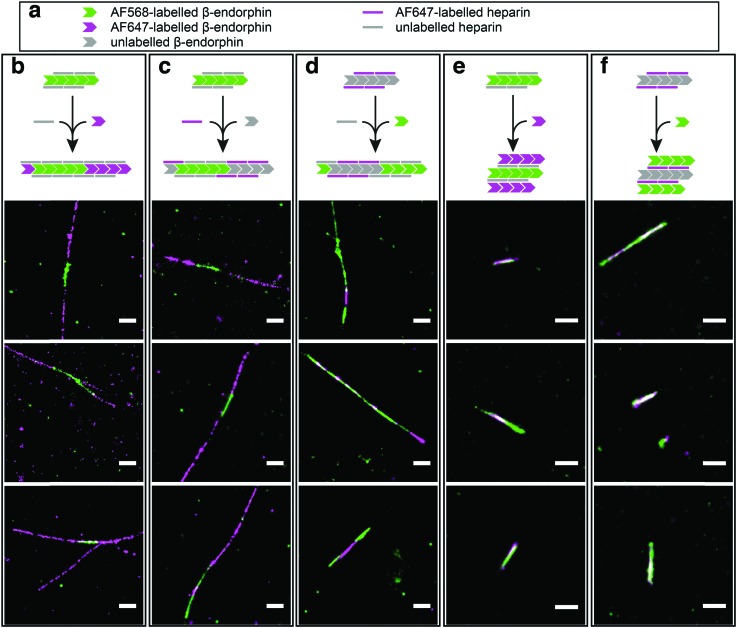
Elongation of heparin-promoted β-endorphin seeds in the presence and absence of heparin. (a) Visual legend to the cartoons illustrating the experimental conditions for each column of data. The chevron and line symbols represent β-endorphin and heparin molecules, respectively. The colours reflect labelling scheme for β-endorphin and heparin: grey – unlabelled, green – labelled with AF568, magenta – labelled with AF647. Panels below the cartoon display representative dSTORM images of the resulting aggregates. The seeds were obtained at 60 μM total peptide concentration including 10% (mol) of labelled β-endorphin and 20 μM heparin. In (b, c and e), seeds formed by AF568-labelled peptide and unlabelled heparin were used for elongation at three different conditions. In (b) AF647-labelled β-endorphin was added to fibril seeds together with unlabelled heparin. Alternatively, unlabelled β-endorphin was added to the seeds together with AF647-labelled heparin in (c). In (e) AF568-labelled seeds were transferred to the solution containing AF647-labelled β-endorphin with no heparin. In conditions (d and f), β-endorphin seeds were formed by the unlabelled peptide and AF647-labelled heparin. Further, these seeds were mixed with AF568-labelled peptide either in the presence (d) or in the absence of heparin (f). Scale bars are 500 nm.

In summary, our experiments demonstrate the coaggregation of heparin and β-endorphin into composite amyloid fibrils. The data obtained *via* two-colour dSTORM explain previously reported striking differences between the properties of heparin-promoted and heparin-free β-endorphin amyloids.^3^ The association of heparin with the peptide leads to a fundamentally different fibril structure that renders the aggregation process irreversible, and, hence, leads to a loss of function of β-endorphin amyloids,^3^ which in nature is required to reversibly interchange between aggregated and monomeric states. Our findings have significant implications for the interpretation of structure–activity relationships and raise concerns on the biological relevance of heparin-promoted amyloid models. The latter is especially relevant for β-endorphin and other functional hormone amyloids since the concentration of heparin in the secretory pathway is insignificant.^12^ Given the widespread adoption of heparin as a universal aggregation promoter,^2,16–20^ the presented findings are quite generally relevant to the field of amyloid research.

This work was funded by grants from the Wellcome Trust, the Medical Research Council UK, the Alzheimer Research UK Trust, the Engineering and Physical Sciences Research Council UK, and the Biotechnology and Biological Sciences Research Council. NN was supported through Early PostDoc.Mobility personal fellowship from Swiss National Science Foundation.
