# Supplementary material for: Heparin acts as a structural component of β-endorphin amyloid fibrils rather than a simple aggregation promoter
Source: Chem Commun (Camb). 2016 Dec 21;53(7):1273–6. doi: 10.1039/c6cc09770g (PMC5436042; doi:10.1039/c6cc09770g)
Supplement: Supplementary file 1 [file CC-053-C6CC09770G-s001.pdf]

Electronic Supplementary Material (ESI) for ChemComm.
This journal is © The Royal Society of Chemistry 2016

Supplementary information to: Heparin acts as a structural component of β-endorphin amyloid fibrils rather than a simple aggregation promoter

Nadezhda Nespovitaya, Pierre Mahou, Romain F. Laine, Gabriele S. Kaminski Schierle, Clemens F. Kaminski*

E-mail: cfk23@cam.ac.uk

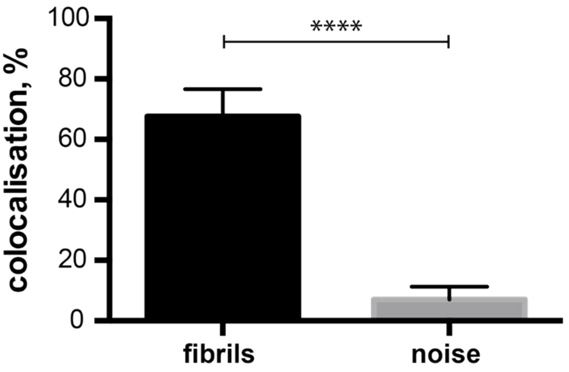

Supplementary Figure S1. Comparative pixel-based analysis of AF647 and AF568 signal colocalisation within fibril clusters (black bar, n=23) and noise (grey bar, n=23). Heparin–promoted β-endorphin fibrils were obtained at 60 μM total peptide concentration including 10 % (mol.) of the AF568-labelled peptide and 20 μM heparin labelled with AF647. Error bars indicate standard deviations. **** - P<0.0001

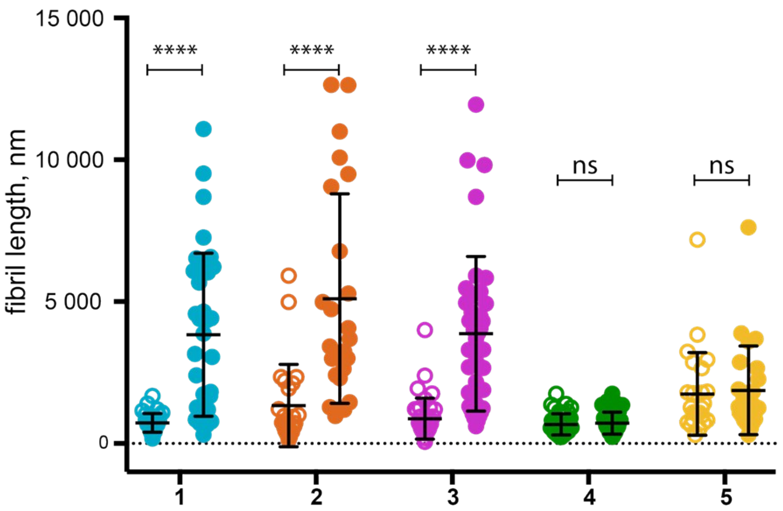

Supplementary Figure S2. Comparative analysis of the length of seeds (open circles) and whole fibrils (filled circles) of heparin-promoted β-endorphin seeds at the end of the elongation assay performed in the presence (**1**, **2**, **3**) and absence of heparin (**4**, **5**). Heparin-promoted β-endorphin fibrils were obtained from the aggregation buffer at 60 μM total peptide concentration and 20 μM heparin. Seeds were obtained by 3-second sonication of fibrils labelled either via β-endorphin with AF568 (**1**, **2**, **4**) or via heparin with AF647 (**3**, **5**). Seeds formed by AF568-labelled peptide and unlabelled heparin (open circles) were used for elongation at three different conditions: (**1**) AF647-labelled β-endorphin was added to fibril seeds together with unlabelled heparin (n=34), alternatively, unlabelled β-endorphin was added to the seeds together with AF647-labelled heparin (**2**, n=24). (**4**) AF568-labelled seeds were transferred to the solution containing AF647-labelled β-endorphin with no heparin (n=40). In conditions (**3**, **5**), β-endorphin seeds were formed by the unlabelled peptide and AF647-labelled heparin. Further, these seeds were mixed with AF568-labelled peptide either in the presence (**3**, n=38) or in the absence of heparin (**5**, n=25). Distributions of fibril length failed D'Agostino-Pearson normality test, therefore, fibril length comparison was performed using Mann-Whitney test (ns – not significant difference, **** $P<0.0001$). Medians and interquartile ranges are displayed for each sample. There is significant elongation observed in the case of elongation of heparin-promoted β-endorphin seeds in the presence of heparin (**1**, **2**, **3**). While, there is no significant difference between seed and whole fibril lengths in the case of elongation in the absence of heparin (**4**, **5**).

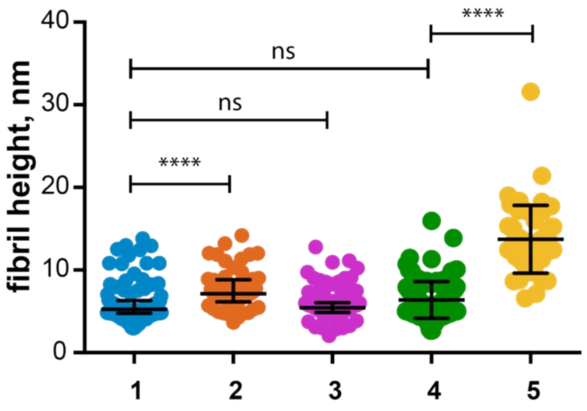

Supplementary Figure S3. Comparative analysis of the height of heparin-promoted β-endorphin seeds (**1**, **4**) before and after the elongation assay performed in the absence (**2**, **5**) and presence of heparin (**3**). Heparin-promoted β-endorphin fibrils were obtained from the aggregation buffer at 60 µM total peptide concentration and 20 µM heparin. Seeds were obtained by 3-second sonication of fibrils labelled either via β-endorphin with AF568 (**1**, shown as blue circles, n = 99) or via heparin with AF647 (**4**, shown as green circles, n = 122). Afterwards, samples were incubated at 37˚C for 48 hours under orbital shaking for equilibration. Next, fresh β-endorphin was added up to 60 µM final concentration including 10 % (mol.) of the peptide labelled with a complementary colour. Mixtures were incubated for 24 hours and imaged with AFM. Fibril heights of the seeds containing AF568-labelled β-endorphin and elongated with AF647-labelled β-endorphin in the absence of heparin (**2**) are shown as red circles (n = 57), the same seeds elongated with AF647-labelled β-endorphin in the presence of heparin are shown as magenta circles (**3**, n=134). β-endorphin seeds containing AF647-labelled heparin (**4**, shown as green circles, n = 122) and elongated with AF568-labelled β-endorphin (**5**, shown as yellow circles, n = 49). For each condition, three AFM samples were prepared and 10 randomly chosen area were imaged. Distributions of fibril height failed D'Agostino-Pearson normality test, therefore, height comparison was performed using Mann-Whitney test (ns – not significant difference, **** P<0.0001). Medians and interquartile ranges are displayed for each sample. There is no significant difference between fibril heights of heparin promoted β-endorphin seeds labelled via the peptide with AF568 (**1**, blue circles, n = 99) or via heparin with AF647 (**4**, green circles, n = 122) and heparin promoted seeds containing AF568-labelled peptide and elongated with AF647-labelled heparin in the presence of heparin (**3**, magenta circles, n=134). However, fibril height changes significantly upon incubation of heparin-promoted seeds with β-endorphin in the absence of heparin (**1** vs. **2** and **4** vs. **5**).

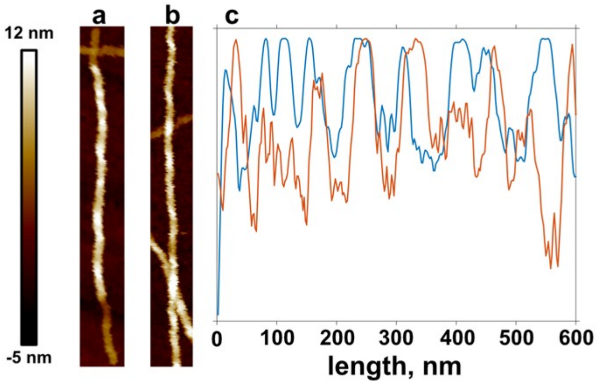

Supplementary Figure S4. AFM data to compare morphology of heparin-promoted β-endorphin seeds elongated in the presence of heparin. (**a**) and (**b**) show representative images of β-endorphin seeds elongated in the presence of heparin. (**c**) Height profile of fibrils shown in (**a**) and (**b**) in blue and red, respectively. Fibrils were formed by co-incubation of 60 µM β-endorphin including 10 % (mol.) of AF568-labelled β-endorphin with 20 µM heparin for 24 hours and sonicated for 3 sec. Further, the obtained seeds were elongated by freshly added 60 µM peptide including 10 % (mol.) AF647-labelled β-endorphin in the presence of 20 µM heparin.

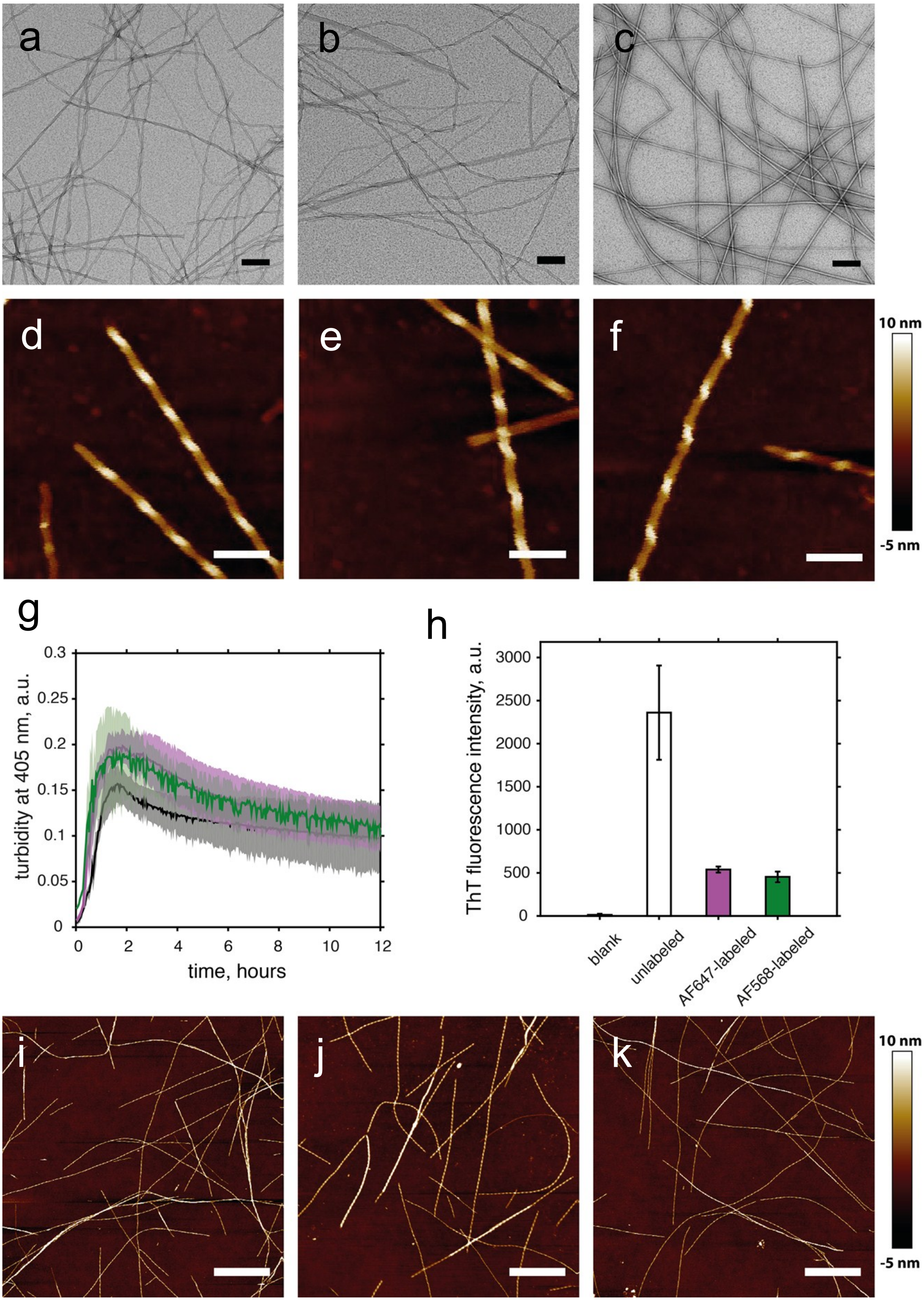
a
b
c
d
e
f
10 nm
-5 nm
g
0.3
0.25
0.2
0.15
0.1
0.05
0
turbidity at 405 nm, a.u.
0
2
4
6
8
10
12
time, hours
h
3000
2500
2000
1500
1000
500
0
ThT fluorescence intensity, a.u.
blank
unlabeled
AF647-labeled
AF568-labeled
i
j
k
10 nm
-5 nm

Supplementary Figure S5. Fluorescent labelling does not affect heparin-promoted aggregation of β-endorphin. Morphology of β-endorphin fibrils obtained in the presence and absence of fluorescent dye labels was studied by EM (**a**-**c**) and AFM (**d**-**f**). β-endorphin fibrils were obtained from the aggregation buffer in the presence of 20 μM heparin at 60 μM total peptide concentration either 100% unlabelled (**c**, **f**) or including 10 % (mol.) of AF647-labelled (**a**, **d**) or AF568-labelled (**b**, **e**) β-endorphin. Fibril morphology of the labelled amyloids (**a**-**b**, **d**-**e**) was very similar to the unlabelled fibrils (**c**, **f**). The scale bar is 250 nm in (**a**-**c**), 200 nm in (**d**-**f**). (**g**) Time-resolved aggregation of β-endorphin in the presence of heparin monitored by absorbance at 405 nm. β-endorphin aggregates were formed by unlabelled β-endorphin (black trace, n = 5), in the presence of 10 % (mol.) AF647-labelled β-endorphin (magenta, n = 5), or in the presence of 10 % (mol.) AF568-labelled β-endorphin (green, n = 4). Error bars represent standard deviations. At the end of the aggregation assay, the resulting mixtures were analysed in the ThT binding assay (**h**). ThT fluorescence intensity was measured for the following samples: blank (black, n = 15), unlabelled β-endorphin fibrils (white, n = 15), β-endorphin fibrils labelled with AF647 (magenta, n = 15), β-endorphin fibrils labelled with AF568 (green, n = 15). Error bars indicate standard deviations. Additionally, the material collected from the aggregation kinetics experiment was imaged by AFM: (**i**) unlabelled β-endorphin fibrils, (**j**) β-endorphin fibrils labelled with AF647, and (**k**) AF568-labelled β-endorphin fibrils. The scale bar is 2 μm (**i**-**k**).

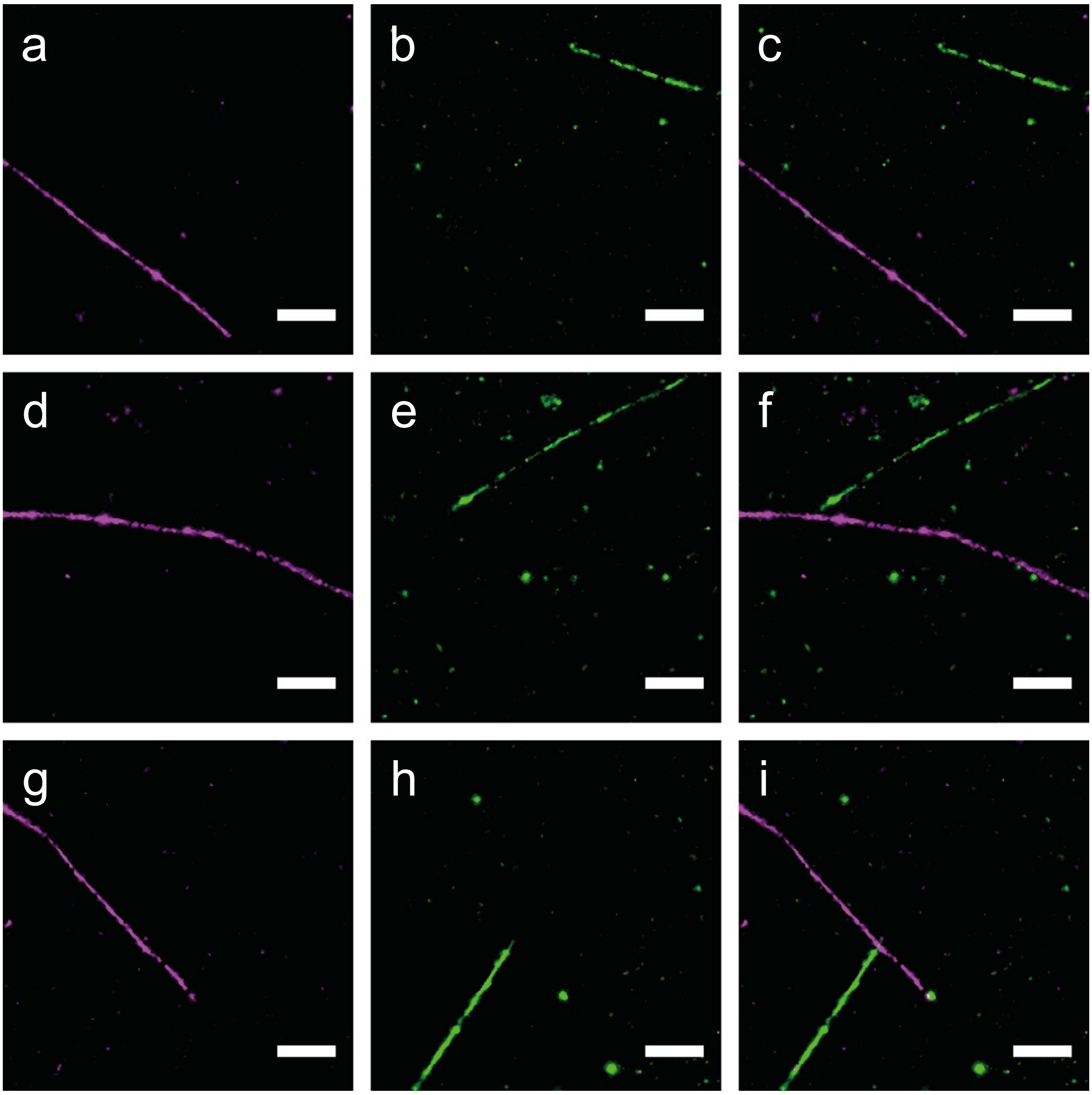

Supplementary Figure S6. Two-colour dSTORM of mature heparin-promoted β-endorphin fibrils labelled with AF647 (magenta) and AF568 (green) mixed together right before imaging. Amyloid fibrils labelled with AF647 and AF568 were obtained separately in the aggregation buffer in the presence of 20 μM heparin at 60 μM total peptide concentration including 10 % (mol.) of the labelled peptide. Each row corresponds to one field of view. Fibril samples were firstly imaged in AF647 detection channel (**a**, **d**, **g**) and then in AF568 channel (**b**, **e**, **h**). Composite two-colour images are shown in (**c**, f, i) where respective super-resolved images are merged. Comparison of panels (**a**, **d**, **g**) against (**b**, **e**, **h**), respectively, demonstrates that there is no spectral bleed-through and AF647 and AF568 dyes are visible only in the corresponding detection channels. Scale bars are 1 μm.

Methods

Peptide expression and purification

All reagents used were purchased from Sigma-Aldrich unless mentioned otherwise. Wild type (WT) β-endorphin was expressed and purified similarly to the method described in [1]. E31C β-endorphin mutant was designed to enable fluorescent labelling of the peptide. All aqueous buffers used for the purification of E31C variant contained 1 mM TCEP (Generon).

Peptide labelling

Two 1-mg aliquots of E31C β-endorphin were dissolved in 2.9 ml of 50 mM Tris-HCl buffer, pH7.0, containing 5 mM TCEP. One mg of Alexa Fluor® 568 C5 and Alexa Fluor® 647 C2 maleimides (Molecular Probes by Thermo Scientific UK LTD) were dissolved in 100 µl DMSO (Thermo Scientific UK LTD) separately. One hundred µl of a dye were added to the peptide solution and mixed gently. Test tubes were covered with aluminium foil and placed on an orbital shaker at room temperature, 75 rpm for 2.5 hours. Two hundred and twenty µl of acetonitrile and 8.5 µl of trifluoroacetic acid (HPLC grade) were added to the mixtures at the end of the labelling reaction. After that, solutions were centrifuged for 5 min at 3500 rpm (rotor 111333, centrifuge Sigma 3-16 PK, SciQuip Ltd). The supernatants were purified on Discovery BIO Wide Pore C18 column (25 cm x 10 mm, 10 µm; Supelco by Sigma-Aldrich Company Ltd.). An Agilent 1260 Infinity HPLC system (Agilent Technologies LDA UK Limited) equipped with an external manual injector, an automatic fraction collector and a diode-array detector was used. The elution profile was monitored by UV absorption at 220, 280, and either 568 or 635 nm. Fractions containing the target peptide were collected, split by 100 µg aliquots, and freeze-dried. Lyophilized labelled peptide was stored at -20 °C until use. The identity and purity of recombinant and fluorescently labelled peptides was confirmed by ESI-MS (Q-TOF II, Micromass).

Heparin labelling

Heparin labelling was performed similarly to the protocol reported earlier by Trindade et al. [2]. Briefly, 1 mg of Alexa Fluor® 647 hydrazide (Molecular Probes by Thermo Scientific UK LTD) was dissolved in 200 µl of 0.1 M HCl (pH preliminary adjusted to 4.8 with NaOH). N-(3-Dimethylaminopropyl)-N'-ethylcarbodiimide hydrochloride (EDAC) was dissolved in 0.1 M HCl, pH 4.8, at 20 mM concentration. Twenty µl of heparin (Heparin sodium salt, Fisher BioReagents, by Thermo Scientific UK LTD) at 50 mg/ml concentration in water, 200 µl Alexa Fluor® 647 hydrazide, and 100 µl of 20 mM EDAC solution were added to 7 ml of 0.1 M HCl, pH 4.8, and stirred. pH of the reaction was continuously monitored and adjusted up to 4.8 when necessary. The labelling reaction was terminated after 40 min by addition of 1.6 ml of 3 M sodium acetate and subsequent incubation under stirring for 40 min more. The reaction mixture was transferred into a dialysis tube (Spectra/Por 3, 3.5 kDa cut-off, Spectrumlabs.com). The dialysis was performed at room temperature and constant stirring for 20 hours.

Fibrillisation

Fibrillisation of β-endorphin was performed at room temperature in Eppendorf Safe-Lock TubesTM (Eppendorf UK Limited) at 37 °C with agitation at 220 rpm in MaxQ4000 benchtop orbital shaker (Thermo Scientific UK LTD). Amyloid fibrils were obtained upon incubation of 60 µM total β-endorphin concentration and 20 µM heparin in 50 mM sodium phosphate, 2 mM sodium citrate buffer, pH 6.0, in the presence of 0.05 % NaN_3 (fibrillisation buffer). Fibrils fluorescently labelled via β-endorphin were obtained by fibrillisation of WT peptide and 10 % (mol.) respectively labelled E31C variant. Amyloid fibrils labelled via heparin were prepared in the presence of AF647-labelled heparin at 20 µM concentration. We confirmed that fluorescent labelling does not affect fibril formation process by time-resolved aggregation assay and microscopy (Supplementary Note 1, Supplementary Fig. S4).

Seeded growth of heparin-promoted β-endorphin fibrils

Heparin-promoted β-endorphin fibrils were prepared as described above. Twenty-four-hour-old fibrils were centrifuged for 3 min at 16,000 × g, 20 ˚C in Heraeus Fresco 21 centrifuge (Thermo Scientific UK LTD). The centrifugation step was intended to eliminate the majority of free β-endorphin

and heparin, however, some of them remained and incorporated into the elongated fibril fragments as evidenced by two-colour dSTORM (Fig. 2b, c). The pellet was resuspended in 250 µl of fibrillisation buffer and sonicated at 10 % power for 3 s by Branson SLPe Digital Sonifier equipped with 4C15 probe (Branson Ultrasonics BV). Afterwards, fibrils were set for incubation for 48 hours at 37 °C with orbital shaking at 220 rpm. In the elongation assay, 100 µl of seeds at 120 µM equivalent monomer concentration were mixed with β-endorphin peptide and heparin when needed. The final reaction volume was kept at 500 µl for all conditions. The final concentration of the added peptide was 60 µM, including 6 µM labelled peptide. Fibril elongation in the presence of heparin was performed at 20 µM heparin concentration. Elongated fibrils were subjected to microscopic studies after 24-hour incubation at 37 °C with agitation at 220 rpm. Statistical analysis of fibril length at the end of the elongation assay was performed using Prism (GraphPad Software, Inc.).

Sample preparation for dSTORM

Prior to imaging, β-endorphin fibrils were centrifuged for 3 min at 16,000 × g. The pellet was diluted by fibrillisation buffer 35 times when fibrils were obtained in the presence of heparin and 10 times after the elongation in the absence of heparin. Two hundred µl of diluted fibrils were incubated on a glass slide (8-well Lab-Tek dishes) for 5 min. After that, unbound fibrils and monomer were gently washed away by the fibrillisation buffer 3-5 times.

dSTORM imaging

The imaging was performed on the inverted total internal reflection fluorescence (TIRF) microscope (Nikon TE-300, Nikon UK Ltd.) custom-built for dSTORM acquisition, as previously described [3,4]. The Nikon TE-300 microscope was equipped with an iXon3 897 EM-CCD camera (Andor). The dyes were irradiated with lasers and their fluorescence collected with appropriate band-pass filters using the following laser-filter combinations: 640nm diode laser (iBeam Smart, Toptica) and 676/37 (Semrock) for AF647, 561/568nm laser (561 DPSS, Oxxius) and 607/ 70 (Semrock) for AF568. For each field of view, a time series of 10,000-15,000 frames per channel was acquired with 10-15 ms exposure and 2–5 kW cm-2 irradiance in the photo-switching buffer (Supplementary Note 2). We verified that there was no crosstalk between fluorescence channels, therefore, fluorescence from AF647 and AF568 dyes can be unequivocally distinguished (Supplementary Fig. S5).

Localisation and super-resolution image reconstruction

All dSTORM data were analysed using rapidSTORM 3.3 [5]. In our study, we achieved a resolution of ~30 nm with AF647 and ~38 nm with AF568 (determined as the full width at half maximum of the localisation error distribution). The resolution was assessed by calculating the full-width half maximum of the localisation precision, from the mean of the localisation precision (standard deviation). The mean of the localisation precision was obtained from its distribution estimated using the formulae presented by Mortensen et al. [6] The chromatic offset between the two fluorescent channels was corrected by applying localisation-based corrections using a custom-written MATLAB routine based on previous work [7]. The chromatic transformations for localisation-based corrections were obtained from white fluorescent microspheres (TetraSpeck microspheres, 0.1 µm, Fisher Scientific UK LTD).

Stimulated emission depletion (STED) microscopy correlated with atomic force microscopy (AFM)

For the correlative STED/AFM imaging in aqueous buffer, we used a home-built STED microscope, as described by Mahou et al. [8]. The super-resolution microscope was equipped with a platform designed for AFM on an inverted optical frame (BioScope Catalyst; Bruker AXS GmbH). Firstly, labelled β-endorphin fibrils were mounted on a high-quality cover glass (PN 474030-9020-000, Carl Zeiss Ltd.) and imaged using STED microscopy over a field of view of 80 × 80 µm2. The excitation wavelength was 640 nm and the depletion wavelength was 765 nm to excite and deplete fluorescence of AlexaFluor 647 (AF647) dye respectively. Then, AFM data were acquired in a selected area of interest within the optical field of view. To ensure correlation between the STED and AFM data, a calibration protocol was used prior each imaging session. STED images were collected

with a pixel size of 20 or 50 nm and a pixel dwell time of 30 µs. AFM data were acquired using PeakForce Quantitative Nanomechanical Property mapping mode with ScanAsyst-Fluid+ probes. The images were acquired at a scan rate of 0.5 Hz and peak force amplitude of 70 nm.

Supplementary Note 1: Effect of fluorescent labelling on formation of heparin-promoted β-endorphin amyloid fibrils

We argue that fibril morphology visualised with high-resolution techniques such as electron microscopy (EM) and AFM combined together with the aggregation kinetics can serve as hallmarks of the amyloid structure, therefore, we use these two properties to assess the influence of the presence of labelled peptide on the amyloid fibril.

Heparin-promoted β-endorphin fibrils were obtained from 50 mM sodium phosphate, 2 mM sodium citrate, pH 6.0 (aggregation buffer), in the presence of 20 µM heparin. The total peptide concentration used was 60 µM, which includes 10 % (mol.) fluorescently labelled peptide. In preliminary experiments, we determined that 10 % fluorescent labelling density is sufficient to obtain high-quality fluorescent images, therefore, this concentration of fluorescently labelled peptide was used throughout the whole study. Morphology of heparin-promoted β-endorphin fibrils was assessed by EM and AFM after 24-hour aggregation at 37 ˚C with 220 rpm orbital shaking (Supplementary Fig. S4a-f). Both methods confirmed high similarity of heparin-promoted fibrils formed in the presence and absence of AF647 and AF568 fluorescent labels. Under EM, all β-endorphin fibrils appeared as thin, long, very well separated filaments. Comparative AFM analysis identified quasi-periodic fibril twists in all studied samples. Therefore, here, we showed that fibril morphology is not affected by the presence of fluorescently labelled peptide in the aggregation mixture using two independent high-resolution microscopic techniques, EM and AFM.

In order to investigate the aggregation kinetics of the labelled fibrils, and avoid possible interference between a typical amyloid stain, such as thioflavin T (ThT), and AF labels, we carried out a turbidity assay in 96-well plate [1], using a wavelength of 405 nm in order to minimise absorbance by AF568 and AF647. Supplementary Fig. S4g shows that heparin-promoted aggregation of β-endorphin was not measurably perturbed by the presence of fluorescently labelled peptide. In all studied cases, no lag phase was observed and an exponential growth phase began immediately after addition of heparin to the reaction mixture. The exponential growth was observed for approximately 2 hours, and then after the maximum was reached the absorbance declined steadily over 10 hours to 50-70 % of the maximal value, similar to what was reported previously [1].

Since turbidity does not distinguish the nature of the aggregates obtained, we collected the aggregates from the 96-well plates and examined their morphology with AFM and stained them with ThT (Supplementary Fig. S4h-k). AFM imaging confirmed fibrillar morphology of all β-endorphin aggregates. Also, the significant increase in ThT fluorescence upon binding to the aggregates collected from the 96-well plate reported the presence of the amyloid structure.

Thus, we demonstrated a negligible effect of the presence of 10% fluorescently labelled peptide on the amyloid formation by β-endorphin polypeptide in the presence of heparin. Neither the morphology nor aggregation kinetics of the labelled aggregates were different from the control unlabelled sample.

Electron Microscopy

5 µl samples of heparin-promoted β-endorphin fibrils were deposited for 1 min on a previously glow-discharged C300Cu carbon-coated copper grid (EM Resolutions). The grid was then blotted, washed twice in drops of double-distilled water and negatively stained for 15 s with filtered 2 % (w/v) uranyl acetate. Images were acquired with FEI Tecnai G2 electron microscope (FEI) operated at 200 kV.

Atomic Force Microscopy

Heparin-induced β-endorphin fibrils at an equivalent monomer concentration of 2-5 µM were deposited for 30 min on High Performance cover glass (PN 474030-9020-000, Carl Zeiss Ltd.) preliminary cleaned with 1 M KOH and coated with 0.01 % poly-L-Lys. Unbound monomer and fibrils

were rinsed gently 3-5 times by deionized water. Afterwards, the samples were dried under nitrogen flow. AFM imaging was performed on BioScope Catalyst microscope (Bruker AXS GmbH). AFM data were acquired using PeakForce Quantitative Nanomechanical Property mapping mode with ScanAsyst-Air probes (resonance frequency - 70 kHz, spring constant - 0.4 N/m, tip radius - 2 nm, Bruker AXS GmbH). The images were acquired at a scan rate of 0.5 Hz and peak force ≤ 1.5 nN. Peak force amplitude varied: mature heparin-promoted fibrils were imaged at 50-70 nm-peak force amplitude, 120-150 nm peak force amplitude was used for seeds elongated in the absence of heparin. Statistical analysis of fibril height in the elongation assay was performed using Prism (GraphPad Software, Inc.).

Aggregation kinetics in 96-well plates

Three 1-ml stocks of β-endorphin peptide at a total concentration of 60 μM, including 6 μM of the fluorescently labelled peptide, were prepared in the aggregation buffer. These samples and the aggregation buffer as a blank were distributed in the non-binding, clear-bottom, black 96-well plate (cat. no. 655906, from Greiner Bio-One) in five replicas of 190 μl. Heparin was added to each well immediately before sealing the plate to yield the final concentration of 20 μM. The 96-well plate was sealed by AMPLiseal™ Transparent Microplate Sealer (PN 676040 from Greiner Bio-One). Absorbance measurements were performed on an EnVision 2104 Multilabel Plate Reader (PerkinElmer). The temperature was set to +37 and +39 °C for the lower and upper heaters, respectively, to prevent condensation. The absorbance at 405 nm was measured with 50 flashes every 144 s at a fixed focal height of 6 mm. Every absorbance measurement was followed by shaking for 110 s at 500 rpm in orbital mode. This cycle was repeated 300 times.

Steady-state thioflavin T binding assay

Th T was dissolved in double-deionized water and filtered through a 0.2 μm pore size Minisart filter unit (Sartorius Stedium Biotech GmbH). The dye concentration was determined by UV absorbance (ε412nm = 36,000 M-1cm-1). A stock solution at 1.5 mM dye concentration was prepared and stored at -20 °C until use. Fifty μl of aggregates obtained in the 96-well plate were diluted 3-fold in the aggregation buffer and mixed with 5 μl of 1.5 mM ThT solution. ThT fluorescence spectra were recorded on F-4500 FL Hitachi Fluorescence Spectrophotometer controlled by FL Solutions software (Hitachi High-Technologies Corporation). The excitation wavelength was set to 440 nm, and three spectra were acquired for each sample in the range of 470−550 nm utilising 2.5 nm slit for excitation and 5 nm slit for emission. The following settings were applied: scanning rate = 240 nm/s, response = 2 s, PMT voltage = 950 V.

Supplementary Note 2: Photo-switching buffers

Enzymatic stock solution:

5 mg glucose oxidase (from Aspergillus niger, lyophilized powder, Sigma-Aldrich)

0.1 ml 1M Tris-HCl, pH 7.5

0.125 ml 1M KCl

2.25 ml H2O

40 μl 0.5 M tris(2-carboxyethyl)phosphine (TCEP), pH 7.0 (Generon)

10 μl catalase (from Bovine liver, aqueous suspension, Sigma-Aldrich)

2.5 ml glycerol

Photo-Switching Buffer:

400 μl 10% glucose

100 μl 1M β-mercaptoethylamine

50 μl enzymatic solution

600 μl PBS (Life Technologies by Thermo Scientific UK LTD)

7 μl 1M KOH

References:

1 N. Nespovitaya, J. Gath, K. Barylyuk, C. Seuring, B. H. Meier and R. Riek, *J. Am. Chem. Soc.*, 2016, **138**, 846–856.

2 E. S. Trindade, C. Oliver, M. C. Jamur, H. A. O. Rocha, C. R. C. Franco, R. I. Boucas, T. R. Jarrouge, M. A. S. Pinhal, I. L. S. Tersariol, T. C. Gouvea, C. P. Dietrich and H. B. Nader, *J. Cell. Physiol.*, 2008, **217**, 328–337.

3 M. Erdelyi, E. Rees, D. Metcalf, G. S. K. Schierle, L. Dudas, J. Sinko, A. E. Knight and C. F. Kaminski, *Opt. Express*, 2013, **21**, 10978–88.

4 G. S. K. Schierle, S. van de Linde, M. Erdelyi, E. K. Esbjoerner, T. Klein, E. Rees, C. W. Bertoncini, C. M. Dobson, M. Sauer, C. F. Kaminski, G. S. Kaminski Schierle, S. van de Linde, M. Erdelyi, E. K. Esbjörner, T. Klein, E. Rees, C. W. Bertoncini, C. M. Dobson, M. Sauer and C. F. Kaminski, *J. Am. Chem. Soc.*, 2011, **133**, 12902–12905.

5 S. Wolter, A. Löschberger, T. Holm, S. Aufmkolk, M.-C. Dabauvalle, S. Van De Linde, M. Sauer and S. van de Linde, *Nat. Methods*, 2012, **9**, 1040–1.

6 K. I. Mortensen, L. S. Churchman, J. A. Spudich and H. Flyvbjerg, *Nat. Methods*, 2010, **7**, 377–81.

7 P. Annibale, M. Scarselli, M. Greco and A. Radenovic, *Opt. Nanoscopy*, 2012, **1**, 9.

8 P. Mahou, N. Curry, D. Pinotsi, G. Kaminski Schierle and C. Kaminski, in *SPIE BiOS*, eds. J. Enderlein, I. Gregor, Z. K. Gryczynski, R. Erdmann and F. Koberling, International Society for Optics and Photonics, 2015, p. 93310U.

9 N. S. de Groot, T. Parella, F. X. Aviles, J. Vendrell and S. Ventura, *Biophys. J.*, 2007, **92**, 1732–1741.
